# Two-time interval method to circumvent particle image velocimetry dynamic range

**DOI:** 10.1016/j.mex.2022.101725

**Published:** 2022-05-07

**Authors:** Sunil V. Bharadwaj, G.R. Vybhav

**Affiliations:** Engineering Mechanics Unit, Jawaharlal Nehru Centre for Advanced Scientific Research, Bangalore, India

**Keywords:** PIV, Entrainment, Turbulent jets

## Abstract

In the velocity range outside the velocity-dynamic range of a PIV (particle image velocimetry) system, the velocity measurements are known to result in noisy velocity vectors due to erroneous detection of correlation peaks, especially in the low-velocity region. Here a simple method is proposed that uses two different timings of a pulsed laser, in conjunction with a median-test of residuals based on the velocity data, to resolve both low and high velocities in a flow field. This method detects the erroneous vectors and replaces them with the correct vectors via a comparison of two sets of PIV data, procured simultaneously with an appropriate time-delay. The validation of the proposed method is demonstrated via a comparison of the present experimental data with previous experiments and DNS (direct numerical simulations) of a round turbulent jet.

• The method uses two-time intervals to resolve two sets of velocities such that each set falls within the dynamic range of PIV corresponding to the time interval. (Dynamic range heavily depends on time interval).
• A quantity called normalised residual median is defined based on “universal outlier detector” which is used to consolidate the two sets of data.
• The strength of the method lies in its ability to obtain instantaneous entrainment velocity even in an unsteady flow.

## Graphical abstract

**Figure:**
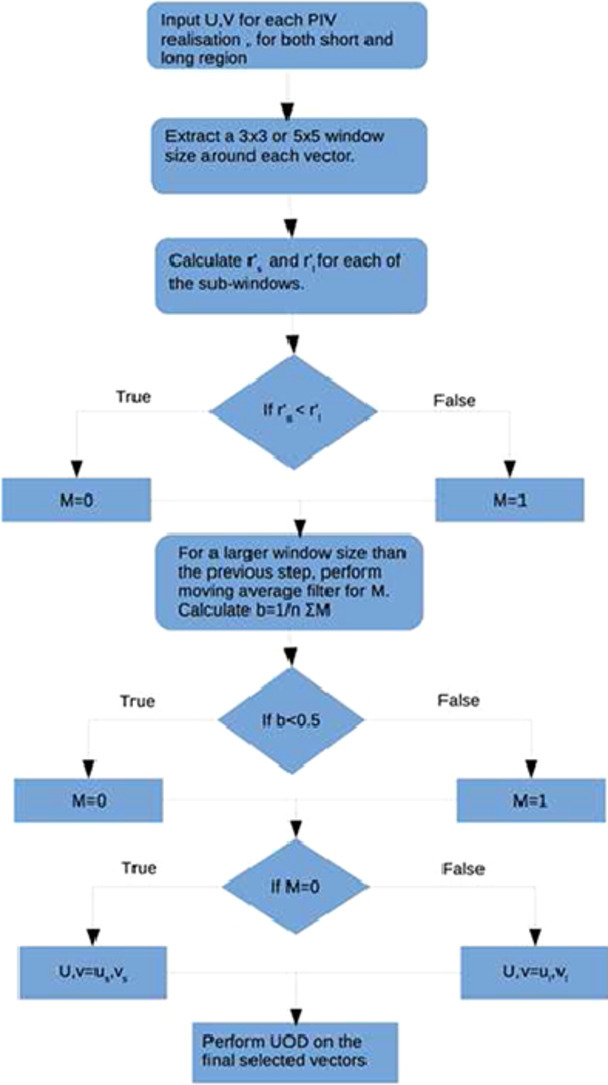

Specifications table

**Table:** 

Subject Area:	Engineering
More specific subject area:	Fluid Mechanics
Method name:	Dual-time Normalised Median Residual Method
Name and reference of original method:	NA
Resource availability:	NA

## Introduction

The resolution of velocity vectors in the slower regions of the flow still remains a challenge in PIV (particle image velocimetry). For example, in a typical jet flow, the jet-core has a high velocity but the ambient, from which the fluid is being entrained, has a relatively low velocity which falls out of the velocity dynamic range (VDR) of the PIV.

In this study, we outline a simple technique in conjunction with the traditional PIV-method, that uses two time-intervals (which is inherent in a pulsed laser), which is able to overcome the problem of resolving low-velocities in the ambient region of a turbulent jet. One of the goals of the PIV (particle image velocimetry) technique is to increase the velocity dynamic range (VDR) which is the ratio of the maximum velocity that a PIV system can measure to the minimum measured velocity [1, 2]. The maximum velocity is usually determined by the interrogation window, but the ratio is a function of the PIV algorithm. Even with improved algorithms incorporating sub-pixel accuracy, the velocity dynamic range currently stands at 100, and Adrian [1] in his review article suggested that techniques/algorithms should be developed such that the VDR can be increased to 1000 to realise the full capabilities of the PIV method, especially for turbulent measurements. The upper limit of the VDR is achieved when the velocities are of the order of the maximum measurable velocity of the PIV system [2], and, therefore, in all practical measurements the VDR achieved is much smaller than the claimed ratio of 100. Recently some researchers have used three laser pulses and a three-point correlation and achieved improved VDR [3], however this requires changes in PIV hardware.

Many outlier detectors are employed in PIV like the median test. Westerweel and Scarano [4] presented an improvement over the original median-test technique of Westerweel [5] in which a “median residual” (see Section 2) is used as an “outlier detector” for spurious velocity vectors of the PIV data — the resulting method seems to work well in a variety of flows (jets, boundary layers, etc.) and hence is dubbed universal outlier detector" (UOD). We instead adopt a different normalisation, “normalised median residual", to detect spurious vectors. With this modified UOD algorithm called the normalised median residual (NMR), we show that both the high and low speed regions of a flow can be simultaneously resolved if the PIV-data is procured using two time-intervals: (i) one resolving the high velocity data and (ii) the other resolving the low velocity data.

In this study, we shall demonstrate the effectiveness of the present method to resolve both the mean and fluctuating velocity fields of a turbulent jet. It may be noted that some experimentalists have used different time-intervals for successive images in PIV to resolve flow fields, albeit in separate runs [6]. The advantage of simultaneous resolution of low-speed and high-speed regions of the flow is crucial in unsteady flows.

## The method: dual-time intervals and a normalized median test

A typical PIV system uses a pulsed laser that illuminates the flow field at desired times synchronised with the camera. A pulsed laser consists of two lasers sharing a laser head, with the time-interval between two laser pulses, Δt, being as low as a few micro-second (μs); the laser itself has a frequency anywhere between 10 Hz and a few kHz. The PIV camera captures a series of images corresponding to the timing diagram shown inFig. 2 in a single run: here two different time intervals arise when the PIV-analysis is performed on images 1 and 2 and 1 and 3 for which the time-intervals are denoted by $\Delta t_{1}$ and $\Delta t_{2}$, respectively, with $\Delta t_{2}$ > $\Delta t_{1}$.

**Fig. 1:**
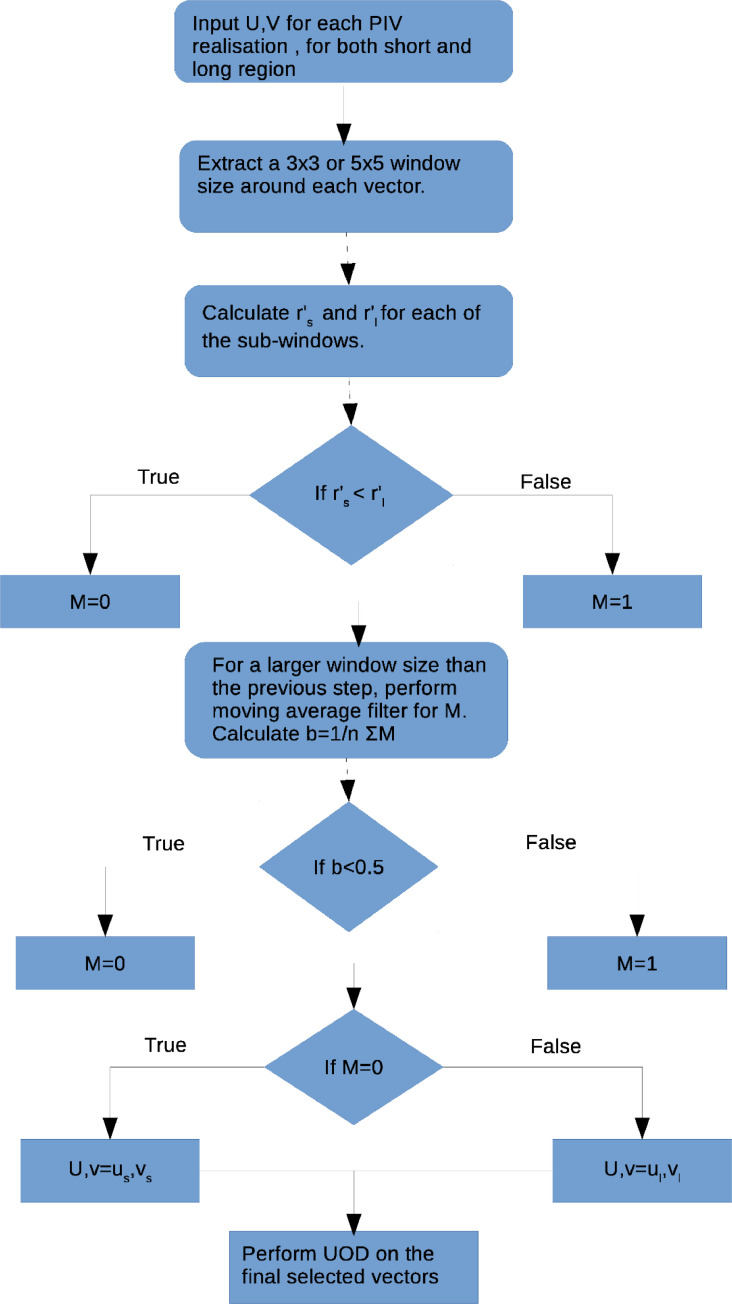
Flow chart of the algorithm. The symbols are referred from the main article. Subscripts “*s*” and “*l*” refer to short and long-time intervals respectively.

**Fig. 2:**
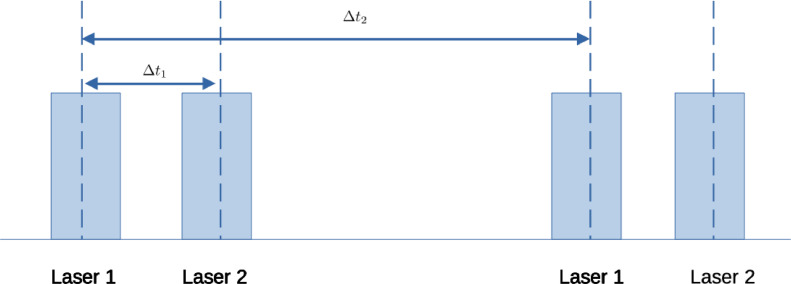
Timing diagram for the pulsed laser: $\Delta t_{1}$ = 1ms and $\Delta t_{2}$ = 100ms.

Every instant of the flow field analysed using PIV results in M × N vectors. A 3 × 3 or 5 × 5 window-size is selected, and the median is calculated.

(1) $$U_{m}=Median\left(U_{1},U_{2},...,U_{n}\right),$$

where *n* = (Window size)^2^, and this procedure is repeated for every vector cell. The residual for each vector, $r_{i}$, and its median, $r_{m}$, are calculated as follows:

(2) $$\begin{array}{c} r_{i}=\left|U_{i}-U_{m}\right|\\ r_{m}=Median\left(r_{i}\right) \end{array}$$

where *i* = 1: *n*. Here, $r_{m}$ is the median residual which we further normalise with $U_{m}$ (the median of the original dataset),

(3) $$r\prime =r_{m}/U_{m}$$

which is dubbed the “normalised median residual”. This quantity (3) will be used to identify spurious vectors.

## Validation

**Fig. 3:**
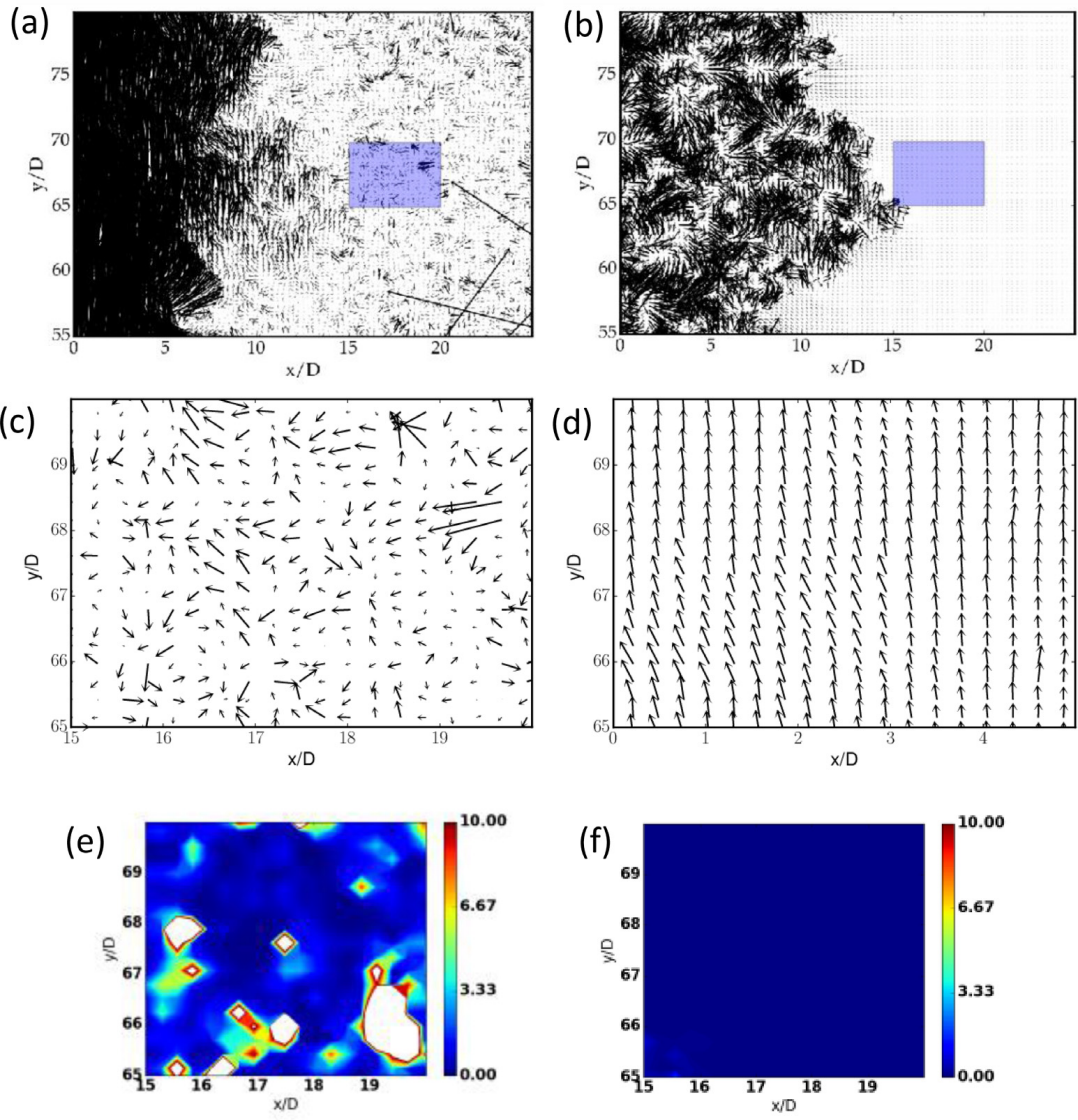
(a, b) Instantaneous velocity-vector maps, with different time-intervals (a) $\Delta t_{1}$. and (b)$\Delta t_{2}$. Panels (c, d) represent zoomed parts of the ambient region of images in panels (a, b) respectively. (e, f) Contour maps of the normalised median residual *r^’^* for panels c ($\Delta t_{2}$) and d ($\Delta t_{2}$), respectively, in the ambient region.

The cross-correlation process of PIV is able to capture the correct magnitudes of velocities within the core-region of a jet when the time-delay is small ($\Delta t_{1}$) - but the same time-delay is likely to yield incorrect magnitude of velocity in the ambient region since there is hardly any movement of the seeding-particles (during $\Delta t_{1}$) due to `low' velocities, see Figs. 3(a,c). On the other hand, the cross-correlation of the PIV would work better in the ambient region if the time delay is made larger ($\Delta t_{2}$ > $\Delta t_{1}$), whereas in the jet-core region the particles are likely to escape the chosen interrogation window (and hence beyond the scope of the algorithm), resulting in spurious velocity vectors in the jet-core region with $\Delta t_{2}$. This is evident in Figs. 3(a,b) which represent the instantaneous vector maps of a turbulent round jet (see Sec. 4 for experimental details) with different time-delays of $\Delta t_{1}$ = 1ms (panel a) and $\Delta t_{2}$ = 100ms (panel b).

In devising the technique, the following ansatz is made: the vectors in the jet-core region (obtained with a short $\Delta t_{1}$) are more correlated than their counterparts obtained with a long $\Delta t_{2}$, while the vectors in the ambient region for long $\Delta t_{2}$ are more correlated compared to the vectors for short $\Delta t_{1}$. The lower correlation results in a higher value of the normalised median residue r′ as defined in Eqn. (3). This implies that the velocities that do not fall within the velocity dynamic range are less-correlated than the ones that do fall in the range. For example, in the ambient region $r_{l}^{i}<r_{s}^{i}$holds on average as evident from Fig. 3(e) and 3(f); here, subscripts *l* and *s* refer to long duration and short duration, respectively. The PIV realisations of both short and long durations ($\Delta t_{1}$ and $\Delta t_{2}$) are compared with the detection criterion. By comparing the respective r′, we can make an appropriate selection between the two Δt.

**Fig. 4:**
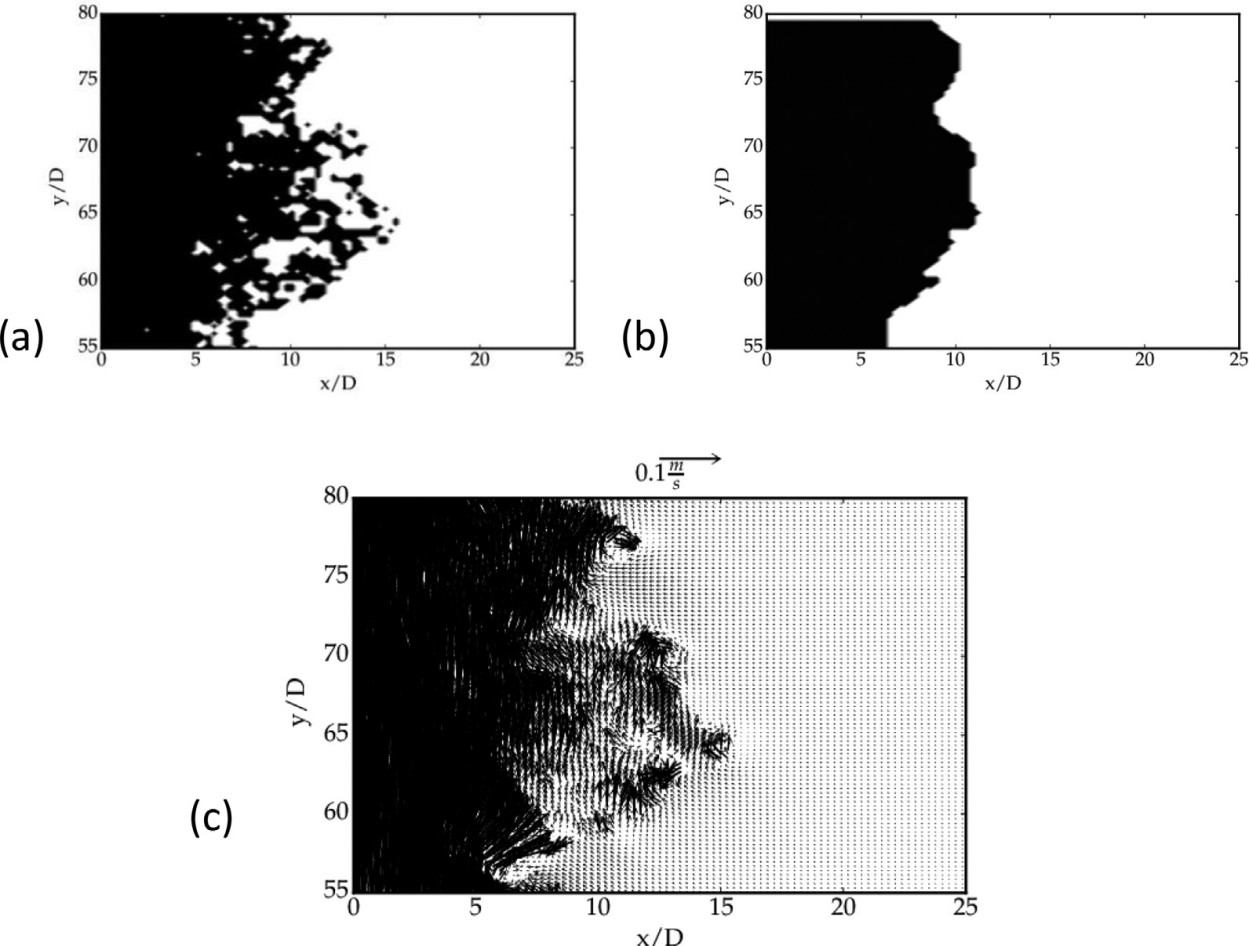
Selection map of the method: (a) unfiltered map, and (b) the filtered maps with a thresholding value of b = 0.5. In each panel, the black dots refer to short $\Delta t=\Delta t_{1}$ representing the high velocity region, the white corresponds to long $\Delta t=\Delta t_{2}$representing the low-velocity region and (c) The resultant vector map after the moving average filter.

It may be noted that the above criterion to select the velocity vectors does not yield a sharp interface between high and low-velocity regions of the jet (see Fig. 4(a)). In particular, although the low-velocity region has been identified by our method, the interface between the jet-core and the ambient region is not well resolved. The latter is resolved by using a moving average filter as described below. Let us assign binary values to the selection map M and calculate the following quantity

(4) $$b=\frac{1}{n}\sum_{1}^{n}M$$

where *M* is the assigned value for selection, i.e. *M* = 1 for long duration ($\Delta t_{2}$) and *M* = 0 for short duration ($\Delta t_{1}$). The above quantity was calculated in a window of 15; ideally, if b > 0.5, the vector takes a value corresponding to the data for long duration, otherwise its value corresponds to data for short duration. Fig. 4(b) indicates that an approximate interface is mapped by the selection between the jet-core and the ambient region. The resultant velocity vector map is shown in Fig. 4(c). The performance of the method at the jet interface is discussed in section 5.

**Fig. 5:**
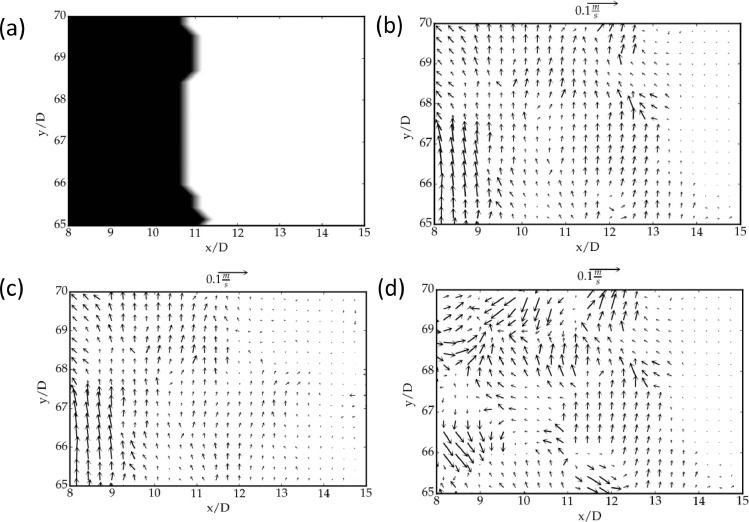
Performance of our method at the interface of selection between $\Delta t_{1}$and $\Delta t_{2}$, i.e. zoomed images of fig 4. (a) Selection map at the interface, colour map same as Fig. 4. (b) The resultant vector map after UOD. (c) Velocity vectors using only t1 after UOD and (d) Velocity vectors using only $\Delta t_{2}$ after UOD.

The method is validated in the case of an axisymmetric turbulent jet. In this section we will show the performance of the method with an emphasis on simultaneous resolution of low-speed and high-speed regions of the flow. Turbulent jet is a canonical example with a vast amount of studied already in literature. In the next section we dwell into more details in the analysis of the flow. The velocity measurements in a circular jet were made over a region spanning from *y* = 55*D* to 80*D* from the nozzle exit for a case of Re ≈ 9400.

## Experimental details

**Fig. 6:**
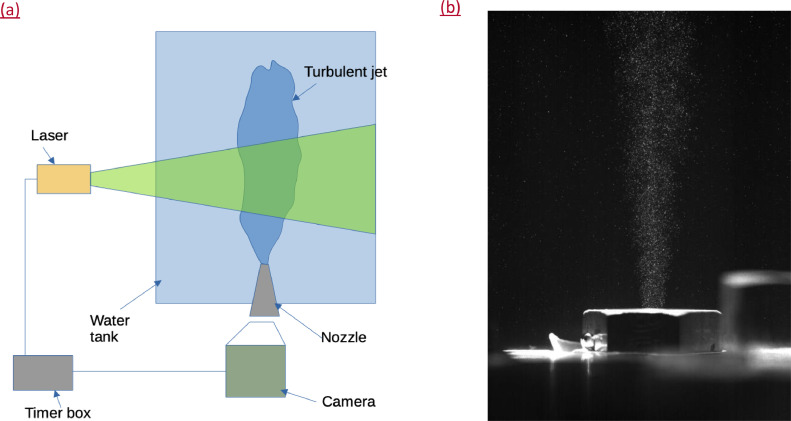
(a) Schematic representing the jet setup along with the PIV system. (b) PIV image at the jet exit; note that the ambient fluid (water) has not been seeded.

Experiments were performed in a glass tank of dimensions 1.5m height and 1m in length and breadth (see Fig 6(a)). The water-jet was issued through a nozzle of exit diameter *D* = 4mm, having a contraction ratio of 11; the flow rate of the jet was adjusted to give a Reynolds number of $ℜ=V_{av}D/\nu \approx 9400$, where ν is the kinematic viscosity of water. For PIV-measurements, the flow field was illuminated by a light-sheet generated using a 150 mJ/pulse, 10 Hz dual-pulsed laser and the hollow glass spheres of diameter of around 30 μm were used as seeding particles. A 4 mega-pixel resolution high-speed camera (IDT Motionpro, 2336 × 1728 pixels) synchronised with laser pulses was used to acquire images which were subsequently processed using the Adaptive Correlation module of the Dynamic Studio software (developed by Dantec Dynamics A/S, Denmark) in multiple steps of the interrogation window of size 32 pixels with 50% overlap. The initial size of the interrogation window was chosen as 128 × 128 pixels and the window size was subsequently reduced to the desired 32 × 32 pixels by using the vectors obtained from the previous step. In the adaptive correlation technique, the information obtained in the previous step greatly reduces the error in PIV vector calculation.

Additionally, to obtain the centreline velocity at the jet exit, we performed experiments close to the jet exit (Fig. 6(b)). The interrogation window used was 32 × 32 pixels with a 75 percent overlap. Due to the high speeds, a Δt of 50μs between laser shot was used. 300 pairs of images were acquired and averaged to obtain the profile at the exit. Due to the limitations of PIV, the first meaningful vectors were obtained at a height of 0.45 *D* from the jet exit.

## Results and discussion

**Fig. 7:**
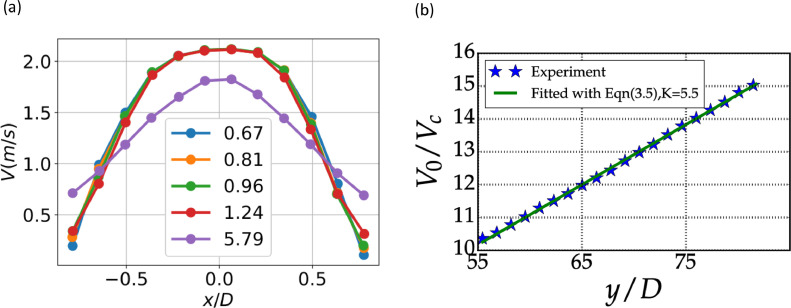
(a) Variations of the axial velocity profiles near the jet-exit. (b) Variation of centreline velocity $V_{c}$ with axial distance. $V_{0}$ is the exit velocity.

The velocity close to the jet exit (at 0.45 D) is shown in Fig. 7. The experiments were performed by replicating the conditions used to perform the experiment at larger axial distance. Despite this, it resulted in small experimental uncertainty. As with the study done by [7] the centreline velocity remains constant close to the exit before decreasing. Xu et al [7] performed experiments with a pipe jet and a contraction jet at Re=86000. They found that for the contraction jet the exit velocity takes a top hat profile. Using the value of jet centreline exit as 2.1 m/s, we assumed a top hat profile (unlike the profile at 0.45 D, which is hardly top hat) and computed the momentum and volume flux at the exit.

The centreline velocity variation with axial distance is shown in Fig. 7(b). The decay of axial velocity of centreline with axial distance is given by

(5) $$\frac{V_{c}}{V_{0}}=K\frac{D}{y-y_{p}}$$

where $V_{c}$ is the centreline axial velocity, $V_{0}$ is the axial velocity at the exit, D is the nozzle diameter, y is the axial location and $y_{p}$ is the axial location of virtual origin, and K is the decay constant. When a straight line is fitted with least squares, the value of K is 5.5. The value of decay constant K is in line with earlier studies, where K varies from 5.5 to 6.7 as suggested by K won et al [8] but falls short of Hussein et al [9] and Bhat and Narasimha [10] who obtained values of 5.8 and 5.7, respectively, but an improvement over Wygnanski and Fiedler [11] who obtained a value of 5.0 for y/D > 50. The smaller value obtained by Wygnanski and Fiedler [11] has been attributed to confinement effects in Hussein's work [9]. The slight disagreement in decay constant in our study too could be attributed to confinement effects. Note that the decay constant is dependent on Reynolds number and the inlet conditions [12].

### Mean flow: axial and radial velocities

#### Comparison with previous experiments

In this section, we will compare results of the consolidated PIV vector maps using the present algorithm with (i) those without the present algorithm (using a commercial software, Dynamic Studio) and (ii) previous experimental results in literature.

In their experiments, Westerweel(2002) [12] and Casey et al (2013) [13] used PIV, whereas Panchapakesan and Lumley(1993) [14] used hot-wire for their measurements. Westerweel(2002) used a closed water tunnel, with a motorised syringe of 1 mm diameter pumping fluid to the main tank and achieved a Reynolds number of 2000. Casey et al (2013) employed a similar setup, opting to use a circular tube of inlet diameter of 5 mm and achieving Reynolds number of 10700. Panchapakesan (1993) [14] used a wind tunnel with an opening of 100mm in a windowless housing around the tunnel and achieved a Reynolds number of 11000. Additionally, we have compared momentum flux decay and centreline decay coefficients with hot-wire measurement performed by Hussein et al [9], Bhat and Narasimha [10], and Wygnanski and Fiedler [11].

**Fig. 8:**
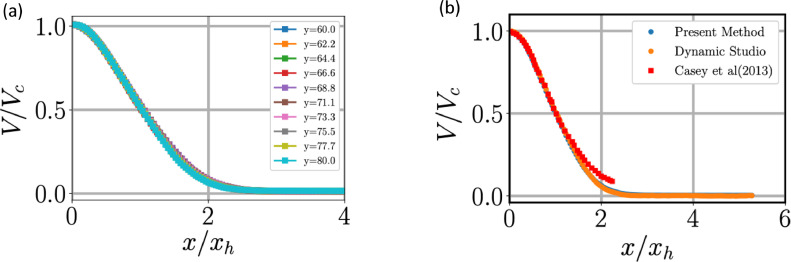
(a) Self-similar profiles of mean axial velocity at different axial heights *y/D*: the local values of the centreline velocity $V_{c}$ and the transverse half-width, $x_{h}$, of jet are used for normalization. (b) Comparison of the present data (at *y/D* = 70) with previous experiments.

**Fig. 9:**
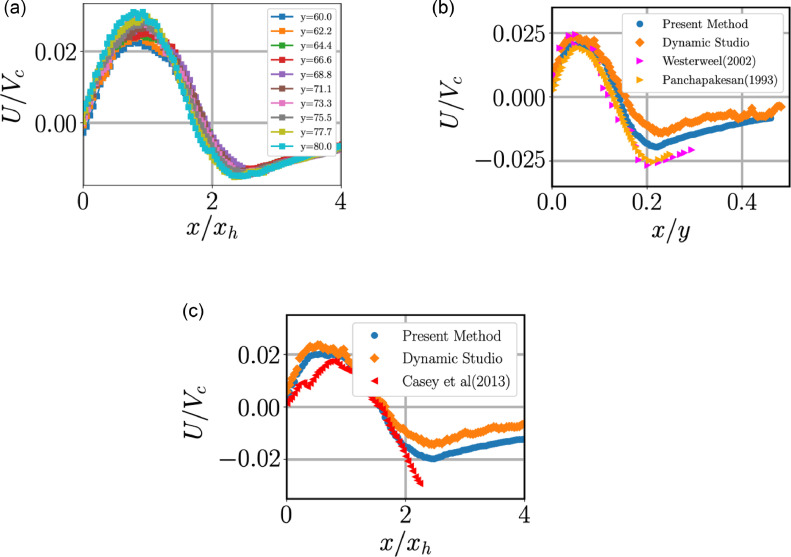
(a) Plots showing self-similar profiles of the radial velocity. (b) Plots comparing conventional PIV with our method for radial velocity and data from [15] and [14]. We have used axial distance to normalise radial distance. (c) Radial velocity plot in comparison with [13]. Notice that our method has yielded a more reliable results at the ambient region of the jet. Quantities are evaluated at *y/D* = 70.

Jets have a self-similar profile when the velocities are normalised with centreline axial velocity and the radial coordinate is normalised either the half width of the jet, $x_{h}$ or the axial distance from the nozzle *y*. In Fig. 8, Fig. 9, we plot the self-similar profiles at different axial distance for mean quantities. In Fig. 8(a), self-similar profiles of axial velocity is plotted where it is clear the lines collapse. However, the radial component (Fig. 9) does not collapse as well as its axial component. This could be due to slight misalignment of the nozzle with the vertical resulting in a small component of axial velocity creeping into the radial component measurements, which can be significant because the axial velocity is around 2 orders of magnitude higher than the radial velocity.

**Fig. 10:**
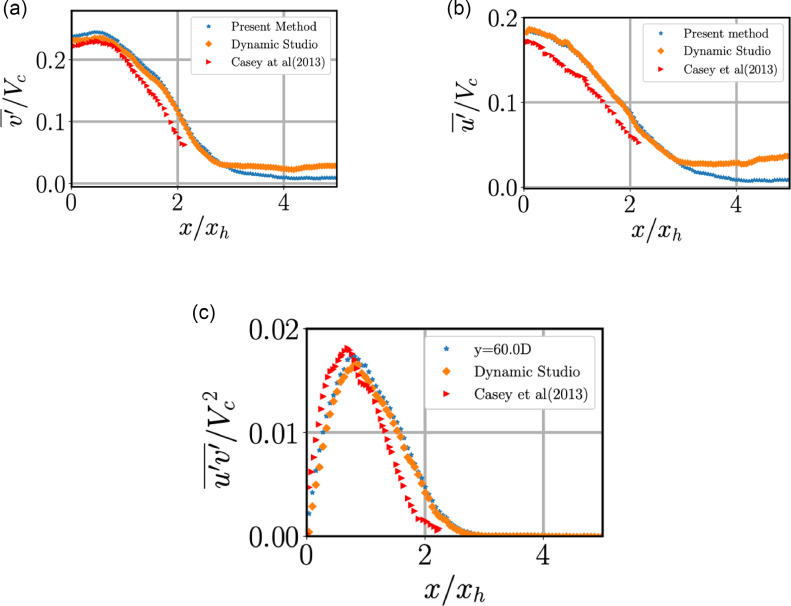
Comparison between conventional PIV and our method for fluctuation velocity and the data from [13]. (a) Axial component of fluctuating velocity, (b) radial fluctuating velocity. (c) One component of the Reynolds stress. Quantities are evaluated at *y* = 70.

The mean and R.M.S velocities are compared between the conventional PIV-algorithm and the method we have used in Figs. 9(b and c) and 10. In panels (b) and (c) we have compared radial velocity with earlier studies but with different normalisations of the radial axis. In both the cases, earlier studies either have truncated their results or did not resolve the ambient like we have achieved. The radial velocity seems to be correctly captured using our method unlike conventional PIV where radial velocity in the ambient is not smooth, which could be attributed to spurious vectors. A lower speed in the ambient region when compared to data from Panchapakesan and Westerweel in Fig. 9(b) could be due to our jet being offset/tilted from the vertical as we could not achieve perfect alignment of the nozzle with the vertical.

#### Comparison with DNS data of van Reeuwijk et al (2016)

van Reeuwijk et al (2016) [16] published their work on jets, plumes and forced plumes with an aim to shed light into entrainment in jets and plumes, along with other findings. We have chosen this study to compare our data with as their study is comprehensive. They chose to normalise the velocities and radial distance with local scales $V_{m}$ and $x_{m}$, respectively, based on integral quantities of the flow defined by

(6) $$x_{m}=\frac{Q\left(y\right)}{M{\left(y\right)}^{1/2}},V_{m}=\frac{M\left(y\right)}{Q\left(y\right)}$$

where the volume flux *Q* and momentum flux *M* is defined by

(7) $$Q\left(y\right)=2\int_{0}^{\infty }Vxdx,M\left(y\right)=2\int_{0}^{\infty }V^{2}xdx$$

**Fig. 11:**
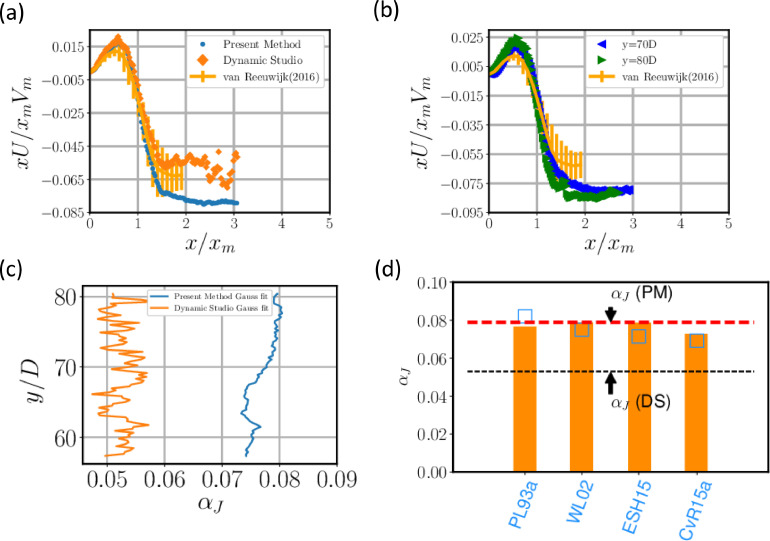
(a) Plot comparing radial velocity with DNS data of [16]. (b) Specific volume flux at *y/D* = 70 in comparison with the DNS data. (c) Entrainment coefficient values with height using our method and Dynamic Studio and (d) Entrainment coefficient values for turbulent jets in previous studies as compiled by van Reeuwijk and Craske [17]. Here PL93A refers to Panchapakesan and Lumley [14], WL02 refers to Wang and Law [18], ESH15 refers to Ezzamel et al [19] and CvR15a refers to Craske and van Reeuwijk [20].

Here, V is the average stream-wise velocity, x is the radial distance from centreline. In other panels we have normalised velocity with centreline velocity ($V_{c}$) and the radial distance with either the jet half width $x_{h}$ or the axial distance from the virtual origin *y*. We use Eqn. (6) to normalise our data which are displayed in Fig. 11. van Reeuwijk et al [16] also suggested a method to calculate entrainment coefficient $\alpha_{J}$ which is not assumed to be a constant a priori. The dilution in jets is calculated using

(8) $$\frac{1}{x_{m}}\frac{dQ}{d\zeta }=-2{\left[xU\right]}_{\infty }$$

where $\zeta =\int_{0}^{y}x_{m}^{-1}dy^{\prime }$. From the entrainment assumption ((Morton, Taylor and Turner 1955), we get

(9) $$-{\left[xU\right]}_{\infty }=\alpha_{J}x_{m}V_{m}$$

where $\alpha_{J}$ is the entrainment coefficient for jets and U is the mean radial velocity. Using Eqn. 9 we evaluate the specific volume flux given by $xU/x_{m}V_{m}$. Outside the jet, in the ambient, we expect the value to be constant which from the equation we can surmise to be the entrainment coefficient $\alpha_{J}$ . To emphasise the importance of resolving the ambient, we plot specific volume ux using our method and conventional PIV (Dynamic Studio) in Fig. 11.

In Fig. 11(b) it is clear that outside the jet the specific volume flux takes more or less a constant value. Using the value of specific volume flux at $x/x_{m}$ = 2, we have evaluated the entrainment coefficient. While using our method, we seem to have obtained a smooth profile of specific volume flux, using conventional PIV the specific volume flux data appears noisy which results in erroneous values of entrainment coefficient as shown in Fig. 11(c). In Fig. 11(d), we plot a figure reproduced from van Reeuwijk and Craske [17] who compiled results from previous studies for entrainment for turbulent jets and plumes. The value of $\alpha_{J}$ agrees very well with the existing studies of turbulent jets. Moreover, due to the resolution of ambient velocities in our flow, we are able to determine $\alpha_{J}$ as a function of axial distance.

## Conclusions

A simple method has been proposed that uses two different timings of a pulsed laser and a method to detect regions with spurious vectors based on the velocity data to resolve low velocities using particle image velocimetry (PIV). The proposed method was able to detect the erroneous vectors and replaced them with the correct vectors obtained from the second set of data that is procured simultaneously using an appropriate time-delay (Δt). This method was validated by carrying out experiments in a round turbulent jet: the radial component of the velocity, which is usually an order of magnitude less than the axial velocity, is successfully resolved even in the ambient region by the present methods compared to that measured by the conventional post-processing techniques adopted in PIV system. The advantage of using this method lies in its potential application in unsteady flows where instantaneous entrainment can be obtained directly from PIV measurements rather than relying on mean-field quantities - which is not possible in an unsteady flow barring ensemble averages consisting of multiple runs.

## Appendix Supplementary materials

Image, application 1
