# Supplementary material for: Two-time interval method to circumvent particle image velocimetry dynamic range
Source: MethodsX. 2022 May 7;9:101725. doi: 10.1016/j.mex.2022.101725 (PMC9127366; doi:10.1016/j.mex.2022.101725)

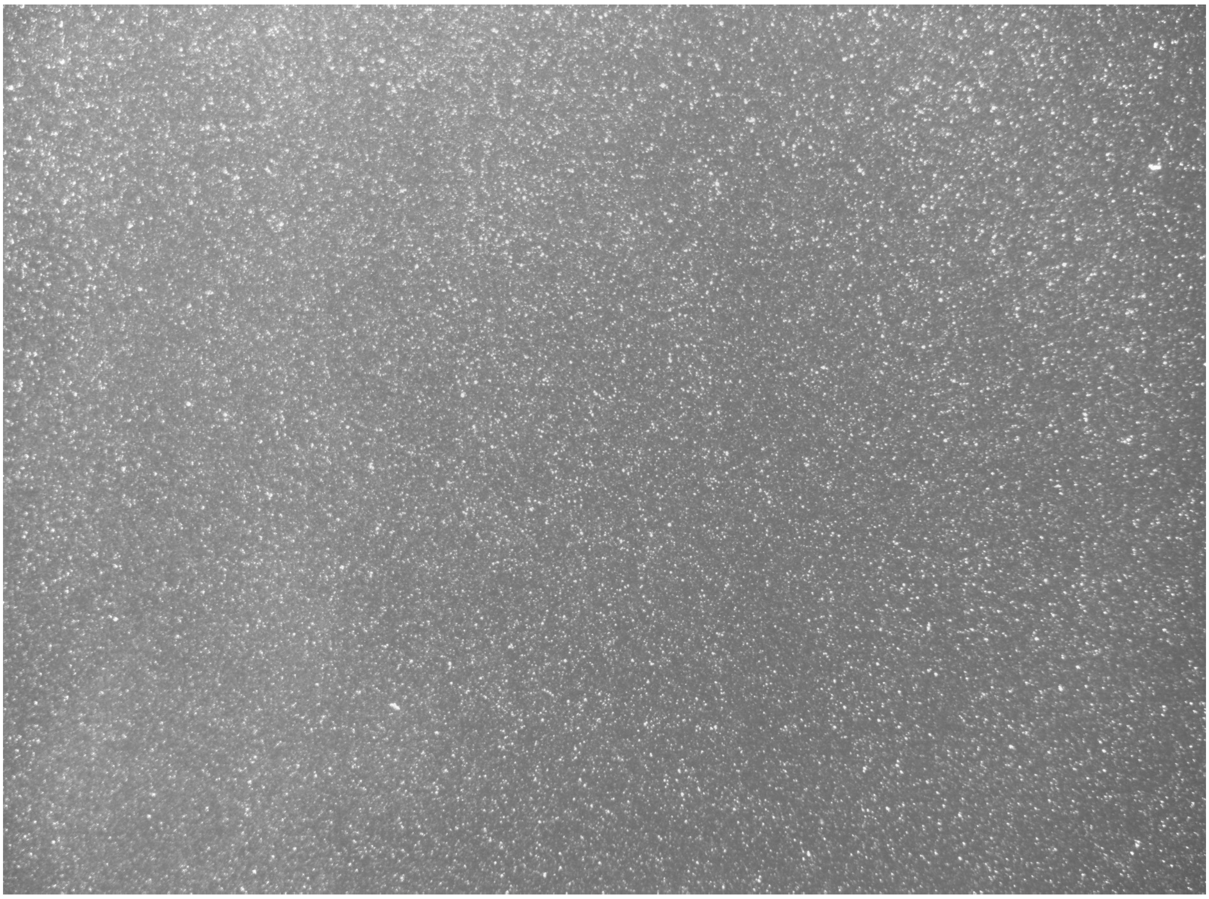

Supplement: Supplementary file 1 [file mmc1.docx]
